# Association of glutathione S-transferase (*GSTM1* and *GSTT1*) genes with chronic myeloid leukemia

**DOI:** 10.1186/s40064-015-0966-y

**Published:** 2015-05-01

**Authors:** Yaya Kassogue, Hind Dehbi, Meryem Quachouh, Asma Quessar, Said Benchekroun, Sellama Nadifi

**Affiliations:** Genetics and Molecular Pathology Laboratory, Medical School of Casablanca, 19 Rue Tarik Ibnou Ziad, Casablanca, BP 9154 Morocco; Department of Onco-Hematology, Ibn Rochd University Hospital, Casablanca, Morocco

**Keywords:** Glutathione S-transferases, *GSTM1*, *GSTT1*, CML

## Abstract

Chronic myeloid leukemia (CML), as most of cancers results from a complex interaction between genetic or non genetic factors. Exposures to xenobiotics endogenous or exogenous associated with a reduced individual ability in detoxifying activity, constitutes a risk of developing cancer. It is known that polymorphism of glutathione S-transferases (GSTs) genes affects the detoxification of xenobiotics. Thus, we conducted a case-control study in which 92 patients (Mean age ± SD, 40.62 ± 12.7 years) with CML and 93 healthy unrelated controls (Mean age ± SD, 41.38 ± 13.4 years) have participated. *GSTM1* and *GSTT1* genotypes were determined by multiplex polymerase chain reaction. Logistic regression was used to assess the possible link between *GSTM1* and *GSTT1* null genotypes and CML as well as between combined genotypes and CML. *GSTM1* null genotype frequency was slightly higher in patients than control (48.9% vs. 40.9%) but, it was not associated with CML (OR 95% CI, 1.4, 0.78-2.48; p = 0.271). Moreover, *GSTT1* null genotype frequency showed a similar trend between patients and control (17.4% vs. 9.7%; OR 95% CI, 1.97, 0.82-4.71; p = 0.13). Surprisingly, *GSTT1* null genotype was significantly associated with the risk of CML in males (OR 95% CI, 5, 1.25-20.1; p = 0.023). The combined *GSTM1* present/*GSTT1* null genotype was found to have a limited effect against the risk of CML (OR 95% CI, 0.3, 0.08-0.99; p = 0.049). Our findings have shown that *GSTT1* null genotype might be a risk factor of CML in males. While, *GSTT1* present genotype might be considered as protective against CML. However, further studies with a large sample size are needed to confirm our findings.

## Introduction

Chronic myeloid leukemia (CML) is a blood cancer secondary to the presence of the reciprocal translocation t(9; 22)(q34; q11) or BCR-ABL gene fusion causing a malignant proliferation of hematopoietic cells (Deininger et al. 2000). However, The etiology of CML like most of cancers, results from a complex interaction between several factors, among others exposure to ionizing and non ionizing radiation, carcinogens present in the environment such as benzene, smoke and pesticides (Salagovic et al. 2000; Gervasini et al. 2007). These factors considered as genotoxics may affect the biotransformation of xenobiotics and cause damage to the DNA of hematopoietic cells and foster the occurrence of CML (Belitsky & Yakubovskaya 2008). Obviously, depending to the genetic constitution on the individual level, the body prevents such damage, thanks to enzymes which play a major role in the activation/detoxification of carcinogens, repair of DNA damage and programming of mutant cells to apoptosis (Belitsky & Yakubovskaya 2008; Hayes and Pulford 1995; Fang et al. 2013). It is noteworthy that hereditary differences which affect an individual’s ability to metabolize xenobiotics represent significant factors in predisposition to develop cancer such as CML (Taioli 1999; Siraj et al. 2008). Glutathione S-transferases (GSTs) are enzymes of phase II, which participate in cellular detoxification by converting active procarcinogenic metabolites of phase I enzymes (cytochrome P450) into inactive metabolites and soluble glutathione, easily execretable. Nowadays, eight classes of GSTs have been identified, including alpha (GSTA), mu (GSTM) theta (GSTT), pi (GSTP), zeta (GSTZ), sigma (GSTS), kappa (GSTK) and omega (GSTO) (Mannervik et al. 1992). Among these, *GSTM1* and *GSTT1* genes which have been mapped on chromosome 1p13.3 and 22q11.2 respectively, are the most studied (Pearson et al. 1993; Webb et al. 1996). It is known that deletions in *GSTM1* and *GSTT1* genes is associated with reduced or absence of enzyme activity (Hallier et al. 1993). Moreover, numerous studies have shown the link between the lack of enzyme activity in *GSTM1* and *GSTT1* and the susceptibility to develop various types of cancer, such as oral cancer, gastric cancer, bladder cancer, and CML in different ethnical groups worldwide (Bajpai et al. 2007; Sharma et al. 2013; Dunna et al. 2013; Ma et al. 2013; Dong et al. 2013; Bhat et al. 2012). Furthermore, other studies have found an association between *GSTM1* and *GSTT1* deletions and the response to chemotherapy, such as disease free survival, overall survival or toxicity (Voso et al. 2002; Mossallam et al. 2006). Given the absence of data on the frequencies of *GSTM1* and *GSTT1* genotypes in our population, we decided to carry out this case-control study with the aim to establish firstly their frequencies in the population and secondly estimate the possible link between the null genotypes and the occurrence of chronic myeloid leukemia in a sample of the Moroccan population.

## Materials and methods

### Subjects

This study was performed after approval of the local Ethics Committee and individual informed consent from patients and control. A total of 92 patients have participated. Patient’s selection was based on the presence of clinically-hematologic signs and Philadelphia chromosome or BCR-ABL gene fusion (Baccarani et al. 2009). Patients were recruited in the department of Onco-Hematology of the Ibn Rochd University Hospital in Casablanca, Morocco from 2011 to 2013. During the same period 93 unrelated healthy controls without a family history of cancer were recruited at the laboratory of genetic and molecular diseases, Faculty of Medicine, the Hassan II University in Casablanca. Four milliliters of venous blood were collected in EDTA tube and kept to minus 20°C till DNA extraction.

### Genotyping of *GSTM1* and *GSTT1* polymorphism

Salting-out method was used to extract DNA from white blood cells (Miller et al. 1988). *GSTM1* and *GSTT1* polymorphism were detected by using a multiplex polymerase chain reaction (PCR) in which BCL2 gene as an internal control was used. The PCR was carried out in a mixture containing 100 ng of genomic DNA, 1X of 5X GoTaq Flexi Buffer (Promega), 1.5 mM of MgCl2, 0.2 mM of each dNTP, 10 pM of each primer and 0.5 U of GoTaq polymerase (Promega) completed to 25 μl with molecular grade water. Forward and reverse primers were 5’-TTCCTTACTGGTCCTCACATCTC-3’ and 5’-TCACCGGATCATGGCCAGCA-3’ respectively, for *GSTT1*; 5'-GAACTCCCTGAAAAGCTAAAGC-3' and 5'- GTTGGGCTCAAATATACGGTGG-3', respectively for *GSTM1*. Forward and reverse primers for BCL2 gene were 5'-GCAATTCCGCATTTAATTCATGG-3' and 5'- GAAACAGGCCACGTAAAGCAAC-3', respectively (Voso et al. 2002). PCR amplification was performed with an initial denaturation at 94°C for 5 minutes, followed by 35 cycles at 94°C for 1 minute, 61°C for 1 minute, 72°C for 1 minute and a last extension at 72°C for 7 minutes. PCR products were analyzed on a 2% agarose gel stained with 0.5 μg/mL ethidium bromide. The *GSTT1*, *GSTM1* and BCL2 produce 480 bp, 219 bp and 154 bp respectively. The presence of BCL2 without *GSTT1* or *GSTM1* reflects their deletion.

### Statistical analysis

Logistic regression was used to assess the risk between *GSTT1* and *GSTM1* null genotypes and the occurrence of CML. Odds ratio (OR) with a confidence interval (CI) of 95% was calculated. The chi-square test was used to compare the genotype distribution between patients and control. *A p-value* less than 0.05 was considered as statistically significant. We have used the statistical package SPSS version 16 (SPSS Inc., Chicago, IL, USA).

## Results

In this case-control study, we explored the frequency of *GSTM1* and *GSTT1* genotypes in 92 CML patients and 93 controls. The patients were composed of 34 males (37%), 58 females (63%) with a mean age of 40.62 ± 12.7; range (18-76 years). However, the control was composed of 39 males (41.9%), 54 females (58.1) with a mean age of 41.38 ± 13.4; range (18-77). The frequencies of *GSTM1* and *GSTT1* genotypes between patients and control are summarized in Table 1. We found that the *GSTM1* null genotype was moderately more frequent in patients (48.9%) compared to control (40.9%), however, this difference was not statistically significant (OR 95% CI, 1.4, 0.78-2.48; p = 0.271). This observation has remained valid for the *GSTT1* null genotype with a frequency of 17.4% in patients against 9.7% in the control group (OR 95% CI, 1.97, 0.82-4.71; p = 0.13) (Table 1). The combination of both polymorphisms shows that subjects carrying the *GSTM1* present/*GSTT1* null were associated with a limited effect (OR 95% CI, 0.3, 0.08-0.99; p = 0.049). Other types of combination showed no trend. The dual deletion which corresponds to *GSTM1* null and *GSTT1* null genotypes has been observed in 6 patients 6.5% against 5 control 5.4% Table 2. Surprisingly, the distribution of participants based on gender revealed that the *GSTT1* null genotype is associated with the development of CML in males (OR 95% CI, 5, 1.25-20.1; p = 0.023) (Table 3). However, in females the *GSTM1* and *GSTT1* null genotypes did not appear to be associated with the occurrence of CML (Table 4).

**Table 1:** **Distribution of** ***GSTM1*** **and** ***GSTT1*** **genotypes between patients and control and evaluation of the risk of CML**

**GST polymorphism**	**Patients**	**Control**	**OR**	**P value**
	**N (%)**	**N (%)**	**95% CI**	
*GSTM1*				
Present	47 (51.1)	55 (59.1)	1	
Null	45 (48.9)	38 (40.9)	1.4 (0.78-2.48)	0.271
*GSTT1*				
Present	76 (82.6)	84 (90.3)	1	
Null	16 (17.4)	9 (9.7)	1.97 (0.82-4.71)	0.13

**Table 2:** **Risk assessment of CML between different combinations of** ***GSTM1*** **and** ***GSTT1*** **genotypes**

***GSTM1***	***GSTT1***	**Patients**	**Control**	**OR**	**P value**
		**N (%)**	**N (%)**	**95% CI**	
Present	Present	37 (40.2)	51 (54.8)	1	
Present	Null	10 (10.9)	4 (4.3)	0.3 (0.08-0.99)	0.049
Null	Present	39 (42.4)	33 (35.5)	0.61 (0.33-1.15)	0.128
Null	Null	6 (6.5)	5 (5.4)	0.61 (0.17-2.13)	0.434

**Table 3:** **Effect of** ***GSTM1*** **and** ***GSTT1*** **genotypes on the development of CML in males**

**GST polymorphism**	**Patients**	**Control**	**OR**	**P value**
	**N (%)**	**N (%)**	**95% CI**	
*GSTM1*				
Present	16 (47.1)	25 (64.1)	1	
Null	18 (52.9)	14 (35.9)	2 (0.79-5.14)	0.145
*GSTT1*				
Present	24 (70.6)	36 (92.3)	1	
Null	10 (29.4)	3 (7.7)	5 (1.25-20.1)	0.023

**Table 4:** **Effect of** ***GSTM1*** **and** ***GSTT1*** **genotypes on the development of CML in females**

**GST polymorphism**	**Patients**	**Control**	**OR**	**P value**
	**N (%)**	**N (%)**	**95% CI**	
*GSTM1*				
Present	31 (53.4)	30 (55.6)	1	
Null	27 (46.6)	24 (44.4)	1.01 (0.52-2.3)	0.823
*GSTT1*				
Present	52 (89.7)	48 (88.9)	1	
Null	6 (10.6)	6 (11.1)	0.9 (0.28-3.06)	0.896

## Discussion

The development of cancer results from an imbalance between exposure to carcinogens (endogenous and exogenous) and the capacity of various enzyme systems engaged in activation or in the detoxification of xenobiotics (Kawajiri et al. 1993). Interindividual genetic variation in xenobiotics metabolizing enzymes has been associated with cancer development (Taningher et al. 1999). Nowadays, many epidemiological studies have shown the implication of *GSTM1* and *GSTT1* polymorphism in tumorigenesis such as CML (Fang et al. 2013; Duggan et al. 2013).

In the current study, the *GSTM1* null genotype frequency (40.9%) was comparable to that observed in Caucasians and Asians (40-62%) but higher than that observed in African-Americans ((16-36%). However, the frequency of *GSTT1* null genotype (9.7%) in our population showed a similar trend to that observed in Caucasians (10-26%) but was less than that observed in Asians (35-52%) (Garte et al. 2001; Van der Logt et al. 2004). The frequency of the homologous deletion (*GSTM1* and *GSTT1* null genotypes), synonymous of absence of enzyme activity was less than that observed in Caucasians (5.4% vs. 10.4%) and that of the Asian population (5.4% vs. 24.6%) (Garte et al. 2001). Trends in variability of *GSTM1* and *GSTT1* null genotypes within a given population might help to estimate the risk of subjects in the development of certain cancers.

In the current study, we noticed that the *GSTM1* null genotype was slightly higher in patients (48.9%) than in control (40.9%); however, it was not associated with an increased risk to develop CML (OR 95% CI, 1.4, 0.78-2.48; p = 0.271) (Table 1). Furthermore, the estimation of CML risk, according to the gender of participants showed similar trends in males (OR 95% CI, 2, 0.79-5.14; p = 0.145) (Table 3) as well as in females (OR 95% CI, 1.01, 0.52-2.3; p = 0.823) (Table 4). Similar findings were observed in India with (OR 95% CI, 1.32, 0.73-2.40; p = 0.4295) (Bhat et al. 2012). Özten et al. 2012, also found no association between *GSTM1* null genotype and CML (OR 95% CI, 1.11; 0.69-1.80; p = 0.714). A meta-analysis realized with a large number of participants confirmed these results (Zintzaras 2009). The analyze of these different results shows that *GSTM1* null genotype seems not to be associated with the development of CML.

Overall, the *GSTT1* null genotype was not found to be associated with the development of CML when males and females were pooled together (OR 95% CI, 1.97; 0.82-4.71; p = 0.13) (Table 1). A study carried in Japan, also failed to find any association between *GSTT1* null genotype and CML (Hishida et al. 2005). Surprisingly, in considering participants according to gender, we have found a significant association between *GSTT1* null genotype and CML in males (OR 95% CI, 5; 1.25-20.1; p = 0.023) (Table 3) but not in females (OR 95% CI, 0.9; 0.28-3.06; p = 0.896) (Table 4). This finding might be explained by differences in xenobiotics exposure between males and females (e.g. Smoke, pesticides) or other genetic/non genetic factors specific to the host. Otherwise, a Turkish study reported an association between *GSTT1* null genotype and CML (OR 95% CI, 2.82; 1.58-5.05; p < 0.001) (Taspinar et al. 2008). Another study done in India, also found that *GSTT1* null genotype was associated with the development of CML (OR 95% CI, 2.67; 1.03-7.01) (Bajpai et al. 2007).

The combined *GSTM1* present/*GSTT1* null genotype was found to have a limited effect against the development of CML when compared to *GSTM1* present/*GSTT1* present genotype (OR 95% CI, 0.3, 0.08-0.99; p = 0.049). Anyway, this finding shows a marginal interaction, between *GSTM1* and *GSTT1* genes. (Zintzaras 2009) has reported a significant association between *GSTM1* present/*GSTT1* null genotype and CML. Elsewhere, we found that the *GSTM1* null/*GSTT1* null genotype was not associated with the development of CML when compared to *GSTM1* present/*GSTT1* present genotype (OR 95% CI, 0.61, 0.17-2.13; p = 0.434). In contrast, other authors have found that *GSTM1* null/*GSTT1* null genotype exposed significantly subjects to develop CML when compared to *GSTM1* present/*GSTT1* present genotype (Bhat et al. 2012; Özten et al. 2012), some authors, reported that subjects with *GSTM1* present/*GSTT1* null genotype were 2.5 times more likely to develop CML when compared to *GSTM1* null/*GSTT1* present genotype (Özten et al. 2012). The results from these authors show a possible interaction between *GSTM1* and *GSTT1*.

## Conclusion

To the best our knowledge, this is the first study on a Moroccan population, assessing the risk of *GSTM1* and *GSTT1* null genotype carriers in the development of CML. This study has allowed to determine the frequency of *GSTM1* and *GSTT1* polymorphism in a sample of our population. In addition, we have noted that the *GSTT1* null genotype is associated with the development of CML in males but not in females.
